# A case report of successful treatment of necrotizing fasciitis using negative pressure wound therapy: Erratum

**DOI:** 10.1097/MD.0000000000014443

**Published:** 2019-02-01

In the article, “A case report of successful treatment of necrotizing fasciitis using negative pressure wound therapy”,^[1]^ which appeared in Volume 98, Issue 2 of *Medicine*, Fabiana Martins de Paula's name appeared incorrectly as Fabiana Martins Paula.
